# EMPT: a sparsity Transformer for EEG-based motor imagery recognition

**DOI:** 10.3389/fnins.2024.1366294

**Published:** 2024-04-18

**Authors:** Ming Liu, Yanbing Liu, Weiyou Shi, Yitai Lou, Yuan Sun, Qi Meng, Dezheng Wang, Fangzhou Xu, Yang Zhang, Lei Zhang, Jiancai Leng

**Affiliations:** ^1^International School for Optoelectronic Engineering, Qilu University of Technology (Shandong Academy of Sciences), Jinan, Shandong, China; ^2^Rehabilitation Center, Qilu Hospital of Shandong University, Jinan, Shandong, China; ^3^Rehabilitation and Physical Therapy Department, Shandong University of Traditional Chinese Medicine Affiliated Hospital, Jinan, Shandong, China; ^4^The Second People’s Hospital of Xintai, Xintai, China

**Keywords:** motor imagery, Transformer, deep learning, self-attention, Mixture of Experts

## Abstract

**Introduction:**

Transformer network is widely emphasized and studied relying on its excellent performance. The self-attention mechanism finds a good solution for feature coding among multiple channels of electroencephalography (EEG) signals. However, using the self-attention mechanism to construct models on EEG data suffers from the problem of the large amount of data required and the complexity of the algorithm.

**Methods:**

We propose a Transformer neural network combined with the addition of Mixture of Experts (MoE) layer and ProbSparse Self-attention mechanism for decoding the time-frequency-spatial domain features from motor imagery (MI) EEG of spinal cord injury patients. The model is named as EEG MoE-Prob-Transformer (EMPT). The common spatial pattern and the modified s-transform method are employed for achieving the time-frequency-spatial features, which are used as feature embeddings to input the improved transformer neural network for feature reconstruction, and then rely on the expert model in the MoE layer for sparsity mapping, and finally output the results through the fully connected layer.

**Results:**

EMPT achieves an accuracy of 95.24% on the MI EEG dataset for patients with spinal cord injury. EMPT has also achieved excellent results in comparative experiments with other state-of-the-art methods.

**Discussion:**

The MoE layer and ProbSparse Self-attention inside the EMPT are subjected to visualisation experiments. The experiments prove that sparsity can be introduced to the Transformer neural network by introducing MoE and kullback-leibler divergence attention pooling mechanism, thereby enhancing its applicability on EEG datasets. A novel deep learning approach is presented for decoding EEG data based on MI.

## 1 Introduction

Motor imagery (MI) brain-computer interface (BCI) systems (MI-BCIs) are designed to help patients with neurological disorders and physical movement disorders to achieve human-computer interaction by transferring the subject’s MI information to the outside world through the communication medium of electroencephalography (EEG) (Hwang et al., 2009; Yao et al., 2014; Shu et al., 2017; Attallah et al., 2020). Changes in subjects’ physical condition and brain activity occur rapidly and can be detected from EEG (Al-Qazzaz et al., 2018). EEG is a non-invasive, safe neurophysiological tool that allows recording brain activities at low cost (Al-Qazzaz et al., 2015). While MI activities are being performed, the subjects are asked to visualize their limb or muscle movements in their brain but not perform actual movements. These cognitive processes cause the relevant brain regions of the brain to be activated thereby generating EEG signals that can be decoded (King et al., 2013).

The study of classification algorithms for MI-EEG signals is an important part of MI-BCIs, and obtaining the subject’s true motor intention through the recognition algorithms is very important for the realization of human-computer interaction or rehabilitation work (Úbeda et al., 2018; Talukdar et al., 2020). Kumar et al. (2017). used a mutual information-based band selection method to utilize all the information obtained from different channels, the features of each frequency band were analyzed using linear discriminant analysis (Kumar et al., 2017). Imran et al. (2014) proposed a discrete wavelet transform method by using time windows to capture the temporal information from EEG, discrete wavelet transform is applied to the data within each window and features are extracted (Imran et al., 2014). The common spatial pattern (CSP) algorithm extracts the temporal features of EEG signals in space for MI tasks by constructing an optimized spatial filter to maximize the variance between the two types of data. Ang et al. (2012) used the filter bank common spatial pattern (FBCSP) algorithm for air domain feature extraction of motion imagery data in frequency bands with good results (Ang et al., 2012).

In recent years, deep neural networks have largely been applied to achieve state-of-the-art performance. Various deep learning models have been successfully employed to decode EEG signals for good performance (Roth et al., 2016; Dutta, 2019; Jiang et al., 2021; Klepl et al., 2022). EEGNet is a compact convolutional neural network consisting of deep and spatio-temporally separated convolutions. It has been used for MI-EEG recognition, showing excellent performance on the BCI competition dataset (Lawhern et al., 2018). Li et al. (2023) proposed a new dual-attention-based MI classification adversarial network MI-DABAN. This network can reduce the distributional differences between domains by analyzing the output differences between two classifiers and can increase the distance between the samples of confusing target domains and the decision boundary to improve the classification performance (Li et al., 2023). Milanés Hermosilla et al. (2021) used the Shallow Convolutional Network to classify and recognize MI-EEG signals with excellent results (Milanés Hermosilla et al., 2021). Kim et al. (2021) investigated different transfer learning strategies and proposed a sequential transfer learning method based on classifier migration, which utilizes the classifier migration technique to sequentially learn the task to improve the execution of MI task efficiency. Due to the difficulty and high cost of acquiring MI-EEG data from patients with central nervous disorders, there have also been studies related to data enhancement and generation of MI-EEG data (Luo and Lu, 2018).

After being proposed by Google in 2017 and achieving superior results in the field of natural language processing (NLP), Transformer neural networks have been migrated to various popular fields and a large number of variants have emerged (Vaswani et al., 2017). All these studies have proved the reliable performance of self-attention mechanism and Transformer neural network. Sun et al. (2022) proposed a parallel Transformer-based and three-dimensional convolutional neural network (3D-CNN) based multi-channel EEG emotion recognition model. The temporal and spatial features of EEG were retrieved by creating parallel channel EEG data and positional reconstruction of EEG sequence data, then using the Transformer and 3D-CNN models (Sun et al., 2022). Wang et al. (2022) proposed variable Transformer to perform hierarchical feature learning of spatial information from electrodes to brain regions to capture spatial information of EEG signals and improve the accuracy of emotion classification tasks (Wang et al., 2022). However, the research on MI-EEG signal recognition is still insufficient (Lee et al., 2021; Ormerod et al., 2021; Singh and Mahmood, 2021; Zhu et al., 2021). The self-attention mechanism for global feature interactions between feature channels is a very effective method for feature extraction, and it has great potential for processing EEG signals because it can capture the global information of the input data very effectively (Xie et al., 2022). However, none of the above work on EEG signal recognition using the Transformer network has been improved for individual differences in samples. The large individual differences in subjects lead to the difficulty of constructing recognition models with generalization to multi-subject MI-EEG data. Transformer networks have the problem of being easily disturbed and difficult to train, which is exacerbated by large individual differences. Adding sparsity structure to the model has become a reliable method to solve this problem. Sparse neural network models can dynamically allocate different depth parameters and structures for different samples or tasks to perform computations. This design allows for the expansion of model width without increasing computational complexity, leveraging the advantages of model scale to avoid a decrease in accuracy caused by individual differences in samples. The effectiveness of sparse models has been validated in various fields. The Extended Transformer Construction introduces strong sparsity to self-attention through the incorporation of Global-local attention, achieving good results in tasks involving long texts and structured inputs (Ainslie et al., 2020). Mustafa et al. (2022) proposed a sparse expert mixture model for multimodal learning, called Language-Image MoE (LIMoE). LIMoE can simultaneously process images and text, and it is trained using contrastive loss. LIMoE has shown performance improvements compared to other models with similar computational complexity across multiple scales (Mustafa et al., 2022). In this study, we add the Mixture of Experts (MoE) and ProbSparse Self-attention mechanism to the Transformer network to increase the sparsity of the model and thus enhance the model’s classification performance on multi-subject data. The concept of MoE was first introduced by Jacobs et al. (1991) to modularize the transformation of multilayer networks. To achieve the goal of expanding the capacity of the model within a limited computational cost, Shazeer et al. (2017) introduced sparse gating networks to MoE, added strong sparsity to the structure of the model and increased the model size by more than 1,000 times at the expense of a very small amount of computational efficiency (Shazeer et al., 2017). Lepikhin et al. (2020) introduced MoE for the first time into the Transformer neural network model, and achieved very good results on the machine translation task with very good results (Lepikhin et al., 2020). To solve the problem of secondary computational complexity of self-attention mechanism, Zhou et al. (2021) proposed the ProbSparse self-attention mechanism, which reduces the memory usage and time complexity for the Transformer model by introducing sparsity (Zhou et al., 2021).

**FIGURE 1:**
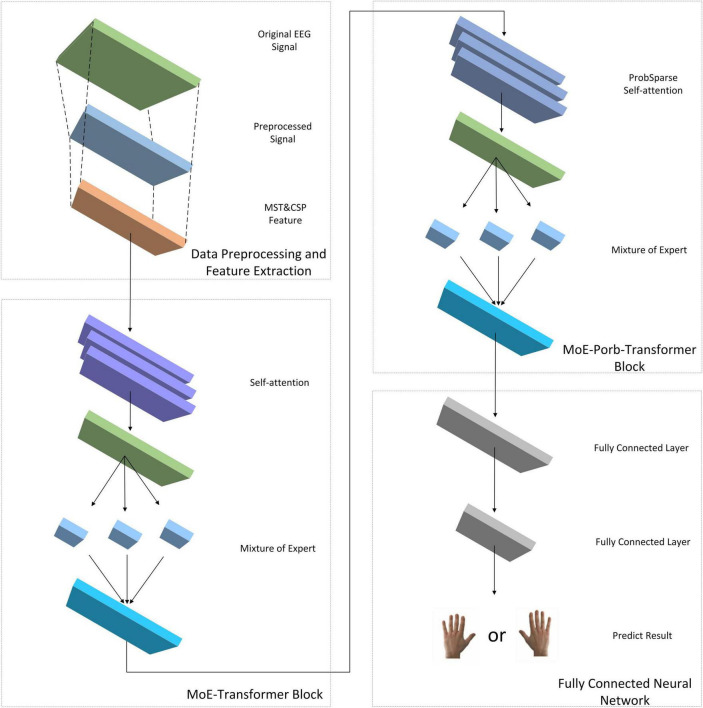
EMPT’s network structure.

This study introduces a Transformer neural network model with the addition of MoE layer and ProbSparse self-attention mechanism for classifying the time-frequency spatial domain features of MI-EEG data of spinal cord injury (SCI) patients, which is named as EEG MoE-Prob-Transformer (EMPT). The model architecture is shown in Figure 1.

The main work of this paper is as follows:

1. The effect of the increase of the MoE layer and ProbSparse self-attention mechanism on the performance of the Transformer structure on EEG data is explored through ablation experiments.
2. The optimal network structure of the EMPT is explored and verified to be effective.
3. The effect of the MoE layer and ProbSparse self-attention mechanism in response to individual differences in subjects are visualized and analyzed to enhance the interpretability of the model structure.

Chapter 2 focuses on the experimental dataset and the main algorithm used in this study. Chapter 3 presents the performance of the Transformer structure on EEG data and the optimal structure of the model with the addition of the MoE layer and the ProbSparse self-attention mechanism. Chapter 4 introduces the visual analysis of the improved parts of the model. Chapter 5 summarizes this study.

## 2 Materials and methods

### 2.1 Dataset

**FIGURE 2:**
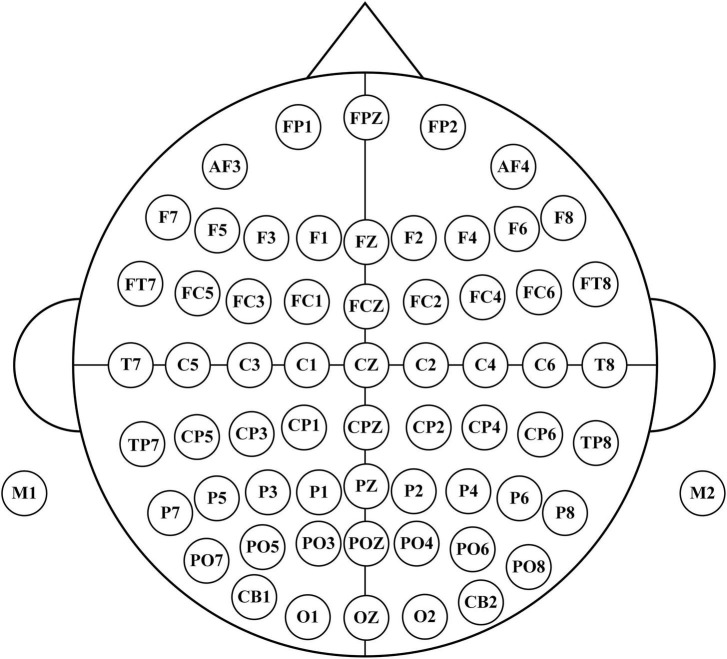
64 electrodes distribution.

**FIGURE 3:**
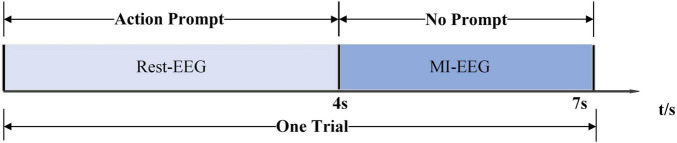
Experimental paradigm.

The dataset was collected from the Department of Physical Medicine and Rehabilitation, Qilu Hospital, Qilu Medical College, Shandong University. All participants provided written informed consent after receiving a detailed description of the purpose and potential risks of the experiment. The study protocol was approved by the Medical Ethics Committee of Qilu Hospital, Qilu Medical College, Shandong University. The experiment was conducted in accordance with relevant guidelines and regulations. The EEG signals were acquired using a 64-electrode acquisition device shown in Figure 2. This dataset was composed of MI-EEG data from 10 subjects (10 SCI patients). During the EEG signal acquisition experiments, the subjects had a complete MI task of 7 s in duration, an imagined movement time of 4 s, and an interval of 3 s between every two imagined movements, and the experimental paradigm is shown in Figure 3. MI tasks are divided into left-handed MI tasks and right-handed MI tasks. The two MI tasks were imagining a left-handed fist clench and a right-handed fist clench. When the MI action cue was over, the subjects started to perform the corresponding MI task. Each experimental group comprised 30 randomly presented MI tasks. Each subject performed 4 groups of experiments with a 90 s rest period between each group of experiments, i.e., each subject performed 4 groups of 120 trials, 60 left-handed MI tasks, and 60 right-handed MI tasks.

### 2.2 Modified S-transform (MST)

The MI-related activity information in EEG signals is mainly concentrated in the alpha band (8–13 Hz) and beta band (13–30 Hz) (Al-Qazzaz et al., 2015; Siddharth et al., 2022). Time-frequency domain analysis of EEG signals has been validated as a very effective method.

Modified S-transform (MST) is a time-frequency domain feature extraction method with independent frequency resolution. MST performs multi-resolution time-frequency analysis of the input EEG data by means of a window function with an adjustable width, which better extracts the phase at different frequencies and clearly locates the frequency profile of the noise. The MST algorithm can optimize the window size and better focus the energy in the time-frequency domain by introducing adaptive parameters (Siddharth et al., 2022).

Modified S-transform (MST) can be expressed as follows,


(1) $$M\,S\,T\,\left(\xi ,f\right)=\int_{-\infty }^{\infty }t\,\left(s\right)\,g\,\left(\xi -s,f\right)\,e^{\left(-j\,2\,\pi \,f\,s\right)}\,dt$$

where *g*(ξ−*s*,*f*) is the Gaussian function of the MST. It is defined as follows,


(2) $$g\,\left(\xi -s,f\right)=\frac{1}{\sqrt{2\,\pi }\,\sigma_{2}\,\left(f\right)}\,e^{\frac{-{\left(\xi -t\right)}^{2}}{2\,\sigma_{2}^{2}\,\left(f\right)}}$$

where the standard deviation σ_2_(*f*) is as follows,


(3) $$\sigma_{2}\,\left(f\right)=\frac{p}{{\left|f\right|}^{q}}$$

The width of the Gaussian window can be optimized by adjusting these two parameters, *P* and *Q*.

The PSD of the MST is calculated as follows,


(4) $$P\,S\,D=E\,\left[M\,S\,T*\bar{M\,S\,T}\right]$$

### 2.3 Common spatial pattern (CSP)

The CSP is employed to find an optimal common spatial filter. After the EEG signals are processed by the optimal spatial filter, the variance of one class of MI-EEG signals is maximized while the variance of the other class of MI-EEG signals is minimized. To obtain the feature vectors with the highest discrimination, the covariance matrices of the CSP for the two classes of MI-EEG signals are diagonalized.

Common spatial pattern (CSP) is able to rely on spatial filters to aggregate the spatial distribution characteristics within the EEG data well and extract the relative spatial information in the signals. Due to its reliability and high computational efficiency, CSP has been widely used for the analysis of EEG signals (Cheng et al., 2016; Fu et al., 2019; Li et al., 2019).

*X*_i_ is the spatio-temporal EEG signaling matrix for the two types of motion imagery The size of *X*_i_ is *C* × *T*_*c*_, where *C* is the number of EEG channels and *T*_*c*_ is the number of time sampling points for each channel.

After normalizing the time-space matrix *X*_i_, the covariance matrix *R*_*i*_ can be obtained as follows,


(5) $$R_{i}=\frac{X_{i}\,X_{i}^{T}}{\text{trace}\,\left(X_{i}\,X_{i}^{T}\right)}\left(i=1,2\right)$$

where $X_{i}^{T}$ denotes the transpose of the matrix *X*_i_, *trace*(*X*)*trace*(*X*) denotes the sum of the elements on the diagonal of the matrix.

The two-class covariance matrix *R* of the mixed space can be defined as follows,


(6) $$R={\bar{R}}_{1}+{\bar{R}}_{2}$$

where ${\bar{R}}_{i}\left(i=1,2\right)$ are the average covariance matrices for task 1 and task 2, respectively.

Since the mixed space covariance matrix *R* is a positive definite matrix, the eigen decomposition is defined as follows,


(7) $$R=U\,\lambda \,U^{T}$$

where *U* is the eigenvector matrix and λ is the diagonal matrix of the corresponding eigenvalues.

These eigenvalues are be arranged in descending order, the transformation *U* can be whitened as follows,


(8) $$P=\frac{1}{\sqrt{\lambda }}\,U^{T}$$

Then *S*_1_ and *S*_2_ can be obtained by the following transformations. *S*_1_ and *S*_2_ have the same eigenvectors.


(9) $$S_{1}=P\,R_{1}\,P^{T},S_{2}=P\,R_{2}\,P^{T}$$

Decompose the principal components of *S*_1_ and *S*_2_ .


(10) $$S_{1}=B\,\lambda_{1}\,B^{T},S_{2}=B\,\lambda_{2}\,B^{T}$$

where λ_1_, λ_2_ are diagonal matrices and the same eigenvector moment *B*.

The sum of the diagonal matrices λ_1_ and λ_2_ of the two eigenvalues is the unit matrix.


(11) $$\lambda_{1}+\lambda_{2}=I$$

The eigenvalues of λ_1_ and λ_2_ are ordered in descending and ascending order, respectively. Since λ_1_ and λ_2_ are the diagonal matrices of *S*_1_ and *S*_2_, for the eigenvector matrix *B*,when *S*_1_ has the largest eigenvalue, *S*_2_ has the smallest eigenvalue. The classification of the two types of MI signals can be achieved by means of the matrix *B*. The projection matrix *W* is calculated as follows.


(12) $$W=B^{T}\,P$$

The projection matrix *W* is the corresponding spatial filter.

### 2.4 Transformer neural network

**FIGURE 4:**
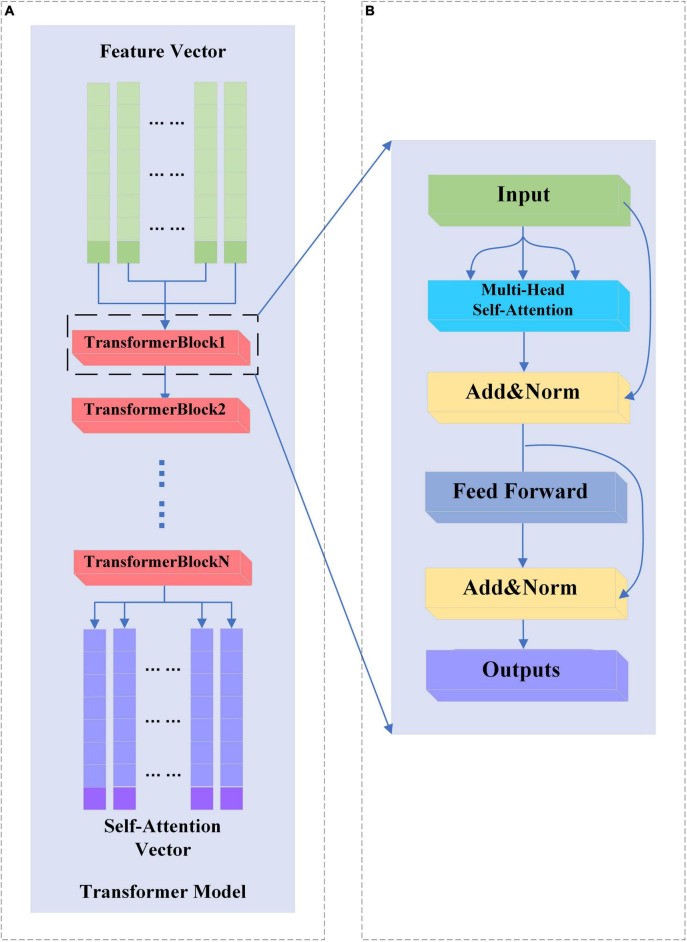
**(A,B)** Transformer and TransformerBlock structure.

In this study, only the encoder structure of the base Transformer network is used. The structure of the baseline Transformer network is shown in Figure 4A. The feature vectors are sequentially entered into several TransformerBlocks thereby being mapped into deep feature vectors containing information about whole brain activity (Han and Wang, 2021).

#### 2.4.1 TransformerBlock structure

A complete TransformerBlock consists of a multi-head attention module, a feed-forward neural network, and an Add&Norm module with corresponding residual connections. The structure of the TransformerBlock is shown in Figure 4B.

The feed-forward neural network in the base Transformer network consists of fully connected layers that rely on a high-dimensional hidden layer transform to map the input vectors and then map the high-dimensional vectors to fixed low-dimensional vectors. This transformation accomplishes deep feature extraction and relies on activation functions to add more nonlinear computation to the network.

The Add&Norm module consists of residual links and layer normalization modules. Its main purpose is to ensure the stability of network training and reduce the occurrence of overfitting phenomenon and network degradation.

#### 2.4.2 Multi-head self-attention

The multi-head attention mechanism consists of multiple mutually independent self-attention heads, each of which can capture different whole-brain activities for reconstructing depth feature vectors. The multi-head attention mechanism expands the sensory field of the attention mechanism for brain activities capture and improves the performance of the attention mechanism.

On the input feature vector*F*, the self-attention module can map three vectors *Q*, *K* and *V* of dimension *L* for computing the attention coefficients of self-attention through the three trainable weight matrices *W^Q^*, *W^V^* and *W^K^*. Where *Q* and *K* are the query vector and key vector, respectively, in the attention mechanism, *Q* and *K* are used to compute the attention dot product, while *V* yields the output vector by weighting with the attention dot product. The formula for *Q*, *V* and *K* calculation is as follows,


(13) $$Q=W^{Q}\,F,K=W^{K}\,F,V=W^{V}\,F$$

The attention factor for *X*_i_ pointing to *X*_j_ is calculated as follows,


(14) $$A_{i,j}=\frac{Q_{i}\,K_{j}}{\sqrt{d_{k}}}$$

(15) $$\sqrt{d_{k}}=\sqrt{L}$$

After obtaining the attention factor matrix *A*_i,j_ for the eigenvector *F*_i_, *V*_i_ is weighted according to the attention coefficient *A*_i,j_. The weighted vector *Z*_i_ is obtained by the following equation.


(16) $$Z_{\text{i}}=\sum_{j=1}^{N}S\,o\,f\,t\,m\,a\,x\,\left(A_{i,j}\right)\,V_{H=j}$$

The self-attention mechanism is used to map the feature vectors of all channels, the original feature vector *F* becomes a new vector containing the attention relations of all feature vectors *Z*. The output vectors of multiple attention heads are spliced together and processed by the feed-forward neural network to be provided to the downstream task.

### 2.5 Sparsity improvement in Transformer networks

Because of the large individual variability of subjects’ EEG signals, when a dataset containing multi-subject data is used to construct a model, a large model width is required to ensure the performance and stability of the model (Suhail et al., 2022). The training samples become larger and each sample needs to go through all the computations of the model, which leads to a large increase in the training cost. In this paper, the MoE layer is introduced to increase the sparsity of the model to save the computational cost. Sparsity means that the model has a large capacity, but only some parts of the model are activated for a single sample. An increase in model sparsity can significantly improve the capacity and performance of a model, but does not proportionately increase the computational effort.

#### 2.5.1 MoE

**FIGURE 5:**
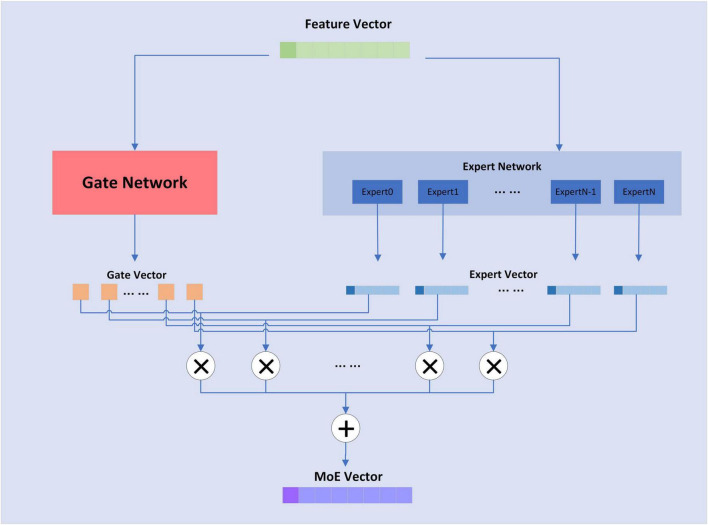
Structure of MoE layer.

The MoE layer has different expert submodels, each specialized for a different input. The experts in each layer are controlled by a gating network that activates certain expert submodels based on the input data. For each input, the gating network selects the most appropriate expert submodel to process the data. The structure of the MoE layer is shown in Figure 5:

The formula for the MoE layer is shown as follows,


(17) $$y=\sum_{i-1}^{n}G_{i}\,\left(x\right)\,E_{i}\,\left(x\right)$$

where *n* is the attribute of the expert sub-model, *G*(*x*) is the output value of the gating network, and *E*(*x*) is the output of the expert sub-model. The composition of the gating network is relatively simple and consists of a linear layer and a softmax activation function, whose formula is shown as follows,


(18) G⁢(x)=S⁢o⁢f⁢t⁢m⁢a⁢x⁢(K⁢e⁢e⁢p⁢T⁢o⁢p⁢K⁢(x⋅W),k)

*KeepTopK*(⋅) is a discrete function that forces values outside of top-k to negative infinity, resulting in an output value of 0 for softmax. For the MoE layer, the expert sub-model is a fully connected layer.

#### 2.5.2 MoE-TransformerBlock

**FIGURE 6:**
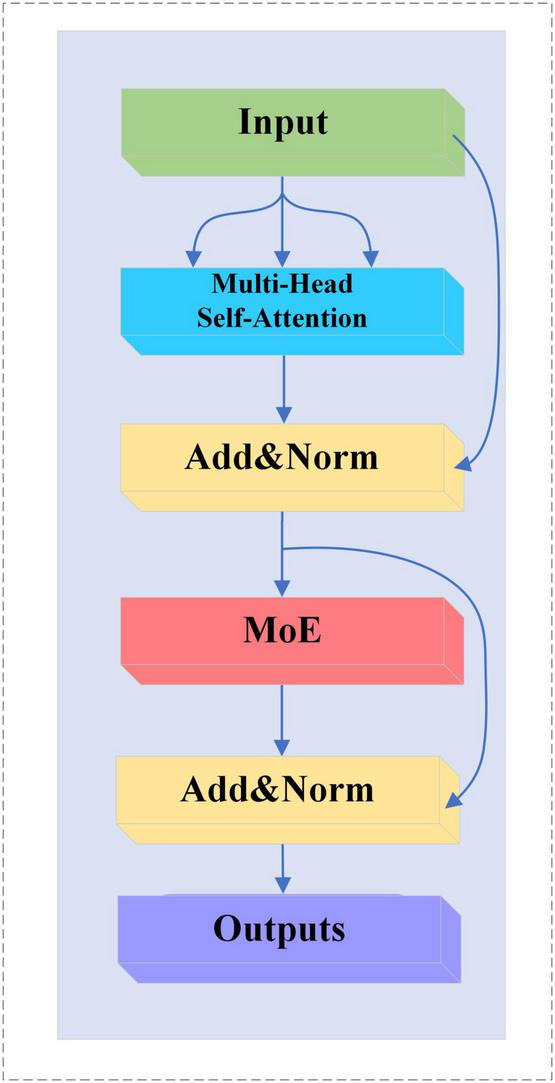
MoE-TransformerBlock structure.

In this study, the feedforward neural network in TransformerBlock is replaced with a MoE layer, which adds sparsity and network width to the model without increasing the computational effort. The MoE-TransformerBlock is shown in Figure 6.

### 2.6 Attention pooling improvements for Transformer networks

**FIGURE 7:**
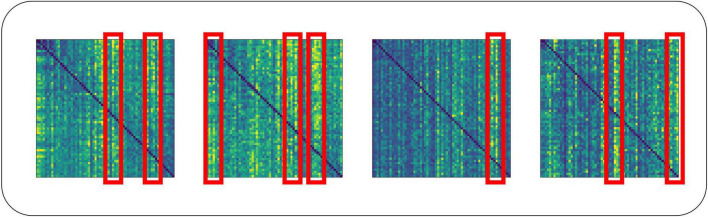
Long-tailed distribution in the dot product of multi-attribute attention.

For the traditional attention mechanism, the dot product of *Q* and *K* is sparse, and the feature map of the self-attention coefficients shows a long-tailed distribution. Long-tailed distribution is a type of uneven data distribution. In a long-tailed distribution, the categories of samples are divided into head and tail categories. The head category means that a few categories contain a large number of samples, and the tail category includes most of all the categories but has only a small number of samples. For a single attention head, fewer dot products contribute the majority of the attention score, and the rest of the paired dot products can be ignored. This sparsity distribution has a practical implication: an element in a sequence will generally only have a high degree of similarity and correlation with a few elements (Zhou et al., 2021). On the EEG dataset, our team similarly confirmed this phenomenon when training the Transformer model, as shown in Figure 7. The head class representation in Figure 7 is boxed in red for easier viewing. For the deeper multi-head self-attention module, the individual attention heads tend to focus more on some specific channels thus showing a long-tailed distribution. This may be because these selected channels already contain the activity of a certain brain region, and the deeper multi-head self-attention module reconstructs the high-level feature vectors that contain the activity of the whole brain by focusing more on these specific channels to capture the global brain activities.

The long-tailed distribution of each set of self-attention coefficients in the traditional self-attention head is similar, and weighting using similar attention coefficients is very wasteful of computational cost. To deepen the stability of the computation and reduce the computational cost, we should find the *Q* that can dominate the distribution of attention coefficients for self-attention computation. To accomplish this, we introduce the ProbSparse self-attention mechanism.

#### 2.6.1 Measuring query sparsity

The long-tailed distribution of the coefficients for traditional self-attention on the EEG dataset is shown in Figure 7. The attention factor of the *i*th query on all keys is defined as the probability *P*(*K*_*H*_,*Q*_*i*_), where H is the number of channels of input EEG features. The probability distribution of the dominant dot product on the attention of the corresponding query is far from the uniform distribution. If *P*(*K*_*H*_,*Q*_*i*_) is close to the uniform distribution, *P*(*K*_*H*_,*Q*_*i*_) = 1/*L*_*K*_, then the query is lazy and fails to pick out important keys, and vice versa, the query is active. If the query is completely lazy, the self-attention becomes a sum of values, which results in some information in the output being redundant.

Since active queries contribute a lot to self-attention and lazy queries contribute little, the active queries are selected as much as possible. The gap between the distribution *P*(*K*_*H*_,*Q*_*i*_) and the uniform distribution can be used to distinguish the importance of a query. ProbSparse self-attention measures similarity by Kullback–Leibler sparsity, the sparsity measurement of the *i*th query is defined as follows,


(19) $$M\,\left(Q_{i},K\right)=\ln\,\sum_{j=1}^{L_{K}}e^{\frac{q_{i}\,k_{j}^{\text{T}}}{\sqrt{d}}}-\frac{1}{L_{K}}\,\sum_{j=1}^{L_{K}}\frac{Q_{i}\,K_{j}^{\text{T}}}{\sqrt{d}}$$

For the sparsity measurement of the *i*th query, the larger the value, the larger the difference between the dot product probability distribution and the uniform distribution, which means the more active the query is.

#### 2.6.2 ProbSparse self-attention

Based on the proposed metric, ProbSparse self-attention is derived by allowing each key to focus on only u main queries.


(20) $$A\,\left(Q,K,V\right)=S\,o\,f\,t\,m\,a\,x\,\left(\frac{\bar{Q}\,K^{\text{T}}}{\sqrt{d}}\right)\,V$$

where $\bar{Q}$ is a sparse matrix of the same size as *Q*, which contains only the Top-u queries under the sparsity metric *M*(*Q*,*K*). For those queries that are not selected, their outputs may be taken as the means of *V*to ensure that both the input and output sequence lengths are *Q*.

Traversing the sparsity measurement *M*(*Q*,*K*) of all queries requires computing each dot-product pair, increasing the quadratic computational complexity *O*(*L*_*Q*_*L*_*K*_), and the log-sum-exp operation has potential numerical stability issues. ProbSparse self-attention uses an empirical approximation that efficiently obtains the query sparsity metric. The improved formula is as follows,


(21) $$M\,\left(Q_{i},K\right)=\underset{j}{m\,a\,x}\left(\frac{Q_{i}\,K_{j}^{\text{T}}}{\sqrt{d}}\right)-\frac{1}{L_{K}}\,\sum_{j=1}^{L_{K}}\frac{Q_{i}\,K_{j}^{\text{T}}}{\sqrt{d}}$$

ProbSparse Attention randomly samples key for each query, the sampling result of each head is the same. However, since each layer of self-attention can do a linear transformation of *Q*, *K*, and *V*, which makes the query and key vectors corresponding to different heads at the same position in the sequence different, so the sparsity measurement of the same query of each head is different, which makes the Top-u query with the highest measurement are different for each head. This is also equivalent to the fact that each head adopts a different optimization strategy.

#### 2.6.3 MoE-Prob-TransformerBlock

**FIGURE 8:**
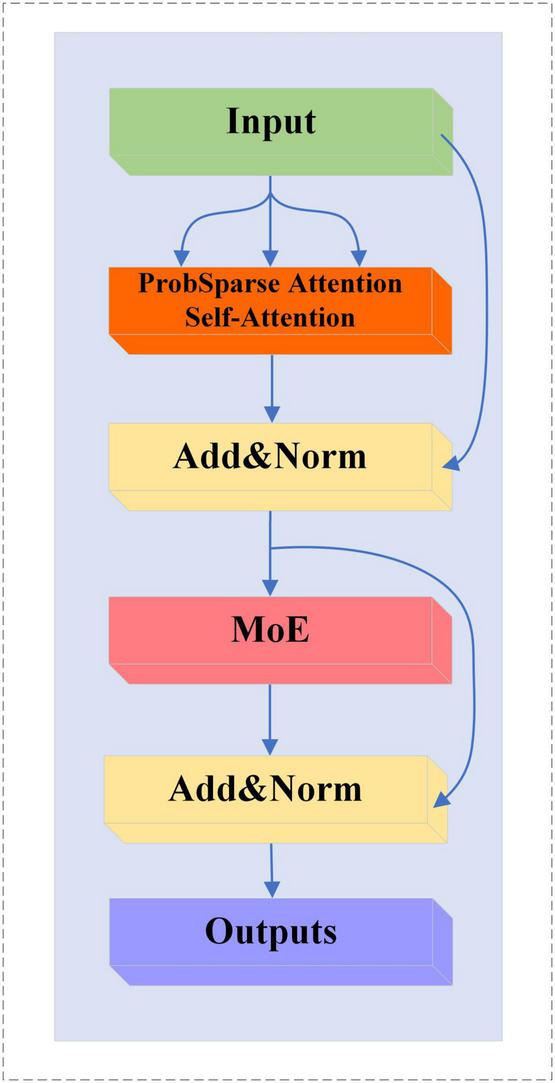
MoE-Prob-TransformerBlock structure.

We replace the multi-head self-attention mechanism in MoE-TransformerBlock with ProbSparse self-attention, the structure of which is shown in Figure 8.

## 3 Results

### 3.1 Implementation details

#### 3.1.1 Pre-processing and feature extraction

For the two different feature extraction methods, this paper uses different preprocessing schemes to MST and CSP on the data.

The shape of the raw EEG data is *T* × *CH* × *ES*, where *T* is the number of experiments, *CH* is the number of channels, and *ES* is the number of sampling points of the EEG signal.

The pre-processing scheme for MST involved passing the raw EEG signals through a Butterworth filter at 8–30 Hz, followed by downsampling. This downsampling step reduced the sampling rate from 1,000 Hz to 100 Hz. After feature extraction by the MST method, the shape of the feature is *T* × *CH* × *Fmst*, where *Fmst* is the number of MST features. The parameters and of the Gaussian window for MST are 0.98 and 0.49.

**FIGURE 9:**
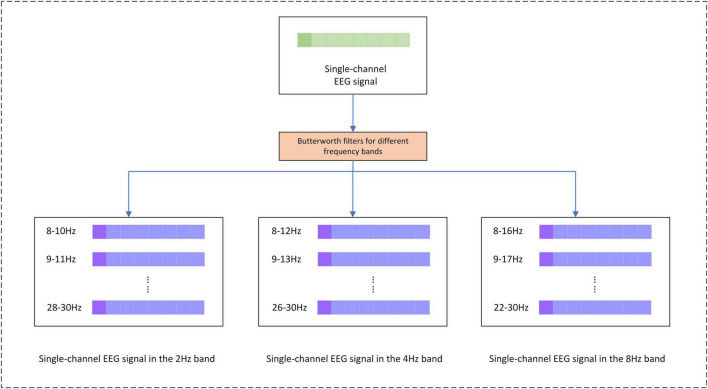
Separation of EEG signals by frequency bands.

In the application of the CSP method, the current study utilizes a multi-band dataset from a single channel for CSP feature extraction. Specifically, the data from multiple frequency bands of each channel is treated as a new channel, and CSP is applied to extract features from these multi-band channels. The pre-processing scheme for the CSP method is as follows, the original EEG signal is decomposed into 55 different frequency bands using a Butterworth filter in windows of band widths of 2, 4, and 8 Hz, all with a step size of 1 Hz (Huang et al., 2009). The signal bands are shown in Figure 9. The shape of the EEG signal data after band decomposition is *T* × *CH* × *FN* × *ES*, where *FN* is the number of frequency bands. After completing the filter decomposition and then downsampling, the sampling rate is reduced from 1,000 to 100 Hz. The EEG signals of each channel are sequentially fed into the CSP method for feature extraction, and the shape of the CSP features is *T* × *CH* × *Fcsp*, where*Fcsp* is the number of CSP features.

#### 3.1.2 Neural network training

**FIGURE 10:**
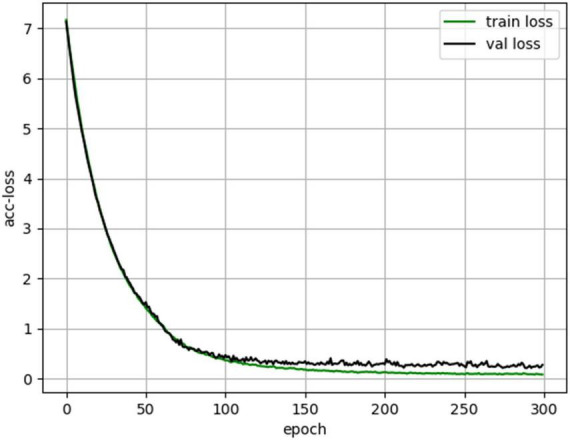
EMPT training loss curves.

The hyperparameters used to train the neural network are shown in Table 1. Where Dropout Rate (FC Layer), Dropout Rate (MoE-TransformerBlock) and Dropout Rate (MoE-Prob-TransformerBlock) are the neuron inactivation probabilities of the fully connected layer, MoE-TransformerBlock and MoE-Prob-TransformerBlock’s neuron inactivation probability. The lower loss rate of MoE-TransformerBlock and MoE-Prob-TransformerBlock is to ensure proper convergence of the model loss. In training, it was found that setting a higher Dropout rate in self-attention leads to too slow convergence of the model loss function. The loss curve for EMPT training is shown in Figure 10.

**TABLE 1:** EMPT model training parameters.

Label	Parameter name	Parameter values
1	Dropout Rate (FC Layer)	0.5
2	Dropout Rate (MoE-TransformerBlock)	0.2
3	Dropout Rate (MoE-Prob-TransformerBlock)	0.2
4	Learn rate	0.00005
5	Batch size	256
6	Epoch	300
7	Multi-head number	8
8	Attention head hidden layer size	128

### 3.2 Experimental results

This section conducts performance experiments and analysis on the EMPT and related structures. Cross-individual model training was performed on the MI-EEG dataset of SCI patients and ten times 10-fold cross validation was performed to obtain experimental results.

#### 3.2.1 Selection of *K* value for MoE layer

In MoE, the *KeepTopK*(⋅) operation selects the larger value *G*(*x*)_*K*_ among the gated network outputs *G*(*x*), with *K* being the number of larger values. expert models corresponding to *G*(*x*)_*K*_ are retained for subsequent weighting operations. expert models with smaller values of *G*(*x*)*G*(*x*) imply that they are not sufficiently important for the current samples. the choice of the value of *K* may be of great significance for the final performance of the model. In order to determine the optimal *K* value for the dataset used in this study, we conducted an experiment to determine the choice of *K* value by looking at the performance of the MoE-Transformer with a layer number of 1 when different *K* values are chosen.

By observing the data within Table 2 we can find that there is little difference in MoE-Transformer performance when *K*≥4. To save unnecessary computational expenses, 4 is chosen as the value of *K* in this study to enable the model to obtain good classification performance.

**TABLE 2:** Performance of single-layer MoE-Transformer at different values of *K*.

*K* value	1	2	4	6	8
accuracy	86.74%	88.43%	89.73%	89.75%	89.88%

#### 3.2.2 Ablation experiment

To verify that the improvements of the MoE layer and ProbSparse self-attention are effective on the SCI EEG dataset, ablation studies are conducted on them separately to explore their effectiveness. The results are shown in Table 3. The MoE-Transformer and Prob-Transformer models are derived by replacing the TransformerBlock with MoE-TransformerBlock and Prob-TransformerBlock based on the Transformer base model. It should be noted that Prob-TransformerBlock is not the MoE-Prob-TransformerBlock described in section “2.6.3 MoE-Prob-TransformerBlock”. Prob-TransformerBlock is obtained by replacing the self-attention in TransformerBlock with ProbSparse self-attention. The experimental results show that the addition of both MoE-TransformerBlock and Prob-TransformerBlock have made improvements to the performance of the Transformer-Base model. From the Table 3, it can be observed that MoE-Transformer and Prob-Transformer still show the best performance at 2 stacked layers for the dataset used compared to Transformer-Base. This may be due to the fact that although both improvements attach strong sparsity to the model to improve performance, both structures do not make the network deeper. The failure of the network to perform better as it gets deeper may also be related to the fact that the dataset used in this paper is not large enough. Although we added sparsity improvements in this chapter to reduce noise interference in the model, due to the noise-sensitive nature of the attention mechanism, smaller datasets still make it difficult to train the model to exclude all noise interference.

**TABLE 3:** Ablation study results.

Model	Block number	Accuracy	Precision	Recall
Transformer-Base	1	88.52%	89.34%	87.68%
	2	93.56%	94.19%	92.38%
	3	90.07%	89.46%	90.67%
	4	86.67%	87.72%	85.63%
	5	85.34%	85.12%	84.88%
MoE-Transformer	1	89.73%	90.52%	88.98%
	2	94.73%	95.68%	93.36%
	3	93.83%	94.26%	93.13%
	4	93.33%	92.52%	93.21%
	5	93.24%	92.19%	93.35%
Prob-Transformer	1	89.23%	89.11%	90.16%
	2	93.85%	92.61%	93.96%
	3	93.13%	93.36%	92.53%
	4	91.67%	92.75%	90.61%
	5	90.62%	91.02%	89.79%

#### 3.2.3 MoE-Prob-Transformer performance experiments

To confirm the optimal stacking order of MoE-TransformerBlock and MoE-Prob-TransformerBlock, this paper conducts comparative experiments for different Block stacking methods to determine the optimal structure of MoE-Prob-Transformer. The results are shown in Table 4, where M stands for MoE-TransformerBlock, P stands for MoE-Prob-TransformerBlock, and FC stands for fully connected layer. Table 4 shows that the stacking method of M-P-FC has the strongest performance. From the experimental results in Table 4, it can be found that when the MoE-Prob-Transformer module is placed more forward, the model’s performance will be lower than the other model stacking methods with the same depth. This phenomenon may be due to the fact that the attention mechanism located in the shallower layer has to aggregate the brain activity information between the channels, so the attention coefficients are less similar, and the use of ProbSparse Attention in the shallower module will result in a loss of brain activity information. However, in the deeper attention module, meaningful brain activities have been aggregated in individual channel features, and a similar long-tailed distribution occurs for the calculation of the attention coefficients. Based on the above analyses, ProbSparse Attention is more suitable to be used at deeper network locations on the SCI MI-EEG dataset. This also explains why Prob-Transformer on Table 3 didn’t get a big boost compared to Transformer-Base.

**TABLE 4:** Experimental results of different stacking structures of EMPT.

Stacking method	Block number	Accuracy	Precision	Recall
M-FC	1	89.73%	90.52%	88.98%
P-FC	1	89.23%	89.11%	90.16%
M-P-FC	2	95.24%	96.38%	94.88%
M-M-FC	2	94.73%	95.68%	93.36%
P-P-FC	2	93.85%	92.61%	93.96%
P-M-FC	2	93.66%	92.82%	94.08%
M-P-P-FC	3	93.22%	92.81%	93.51%
M-M-P-FC	3	94.63%	93.21%	95.43%
M-M-M-FC	3	94.33%	93.26%	94.13%
P-M-M-FC	3	93.27%	92.55%	93.48%
P-P-M-FC	3	92.65%	93.23%	91.32%
P-P-P-FC	3	93.13%	93.36%	92.53%

#### 3.2.4 Comparative experiments

**FIGURE 11:**
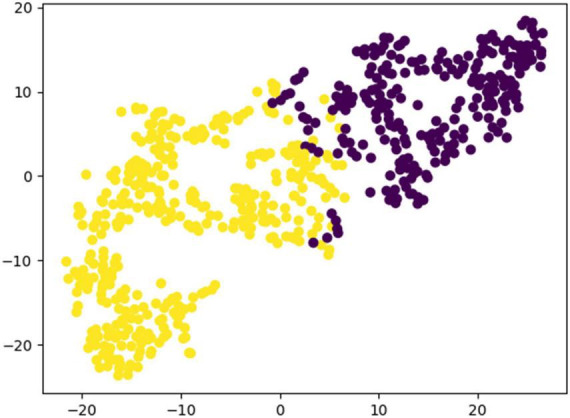
Results of t-SNE visualization for EMPT feature vectors.

To verify the performance of the proposed model, we conducted a comparison test with other state-of-the-art classification models on the same dataset, and the results are shown in Table 5. From Table 5, it can be found that EMPT not only achieves superior performance in comparison with many commonly used methods, but also achieves leading results in comparison experiments with three attention models, attention-based temporal convolutional network (ATC-Net), multi-scale adaptive transformer network (MSATNet), and metric-based spatial filtering transformer (MSFT), which suggests that the model proposed in this study is very effective. To validate the effectiveness of EMPT, we conducted t-distributed stochastic neighbor embedding (t-SNE) visualization of the vectors before entering the fully connected layer. The results are presented in Figure 11. In Figure 11, the purple dots represent EEG trials of the left-handed MI task and the yellow dots represent EEG trials of the right-handed MI task. As depicted in Figure 11, the features after feature decoding by EMPT are separable.

**TABLE 5:** Comparison test results of different models.

Model	Accuracy	Precision	Recall
CWT/PCA+SVM (Bousseta et al., 2016)	86.24%	87.39%	85.22%
EEGnet (Lawhern et al., 2018)	88.73%	87.91%	89.47%
HS-CNN (Dai et al., 2020)	89.36%	90.27%	89.34%
CNN+LSTM (Amin et al., 2022)	90.21%	89.32%	90.45%
ATC-Net (Altaheri et al., 2023)	92.44%	91.62%	93.33%
MSATNet (Hu L. et al., 2023)	93.59%	94.45%	93.18%
MSFT (Jia et al., 2023)	94.18%	94.74%	93.69%
EMPT	95.24%	96.38%	94.88%

To verify the model performance of EMPT, we compared it with the state-of-the-art models on the BCI competition dataset IV-2A, and the experimental results are shown in Table 6. The experimental results prove that EMPT also performs well on the BCI competition dataset IV-2A.

**TABLE 6:** The performance on the BCI competition datasets IV-2A.

Method	Subjects									
	**A01**	**A02**	**A03**	**A04**	**A05**	**A06**	**A07**	**A08**	**A09**	**AVG**
EEGNet (Lawhern et al., 2018)	83.68	63.89	90.97	64.24	59.72	52.08	87.85	82.29	86.81	74.61
MI-DABAN (Li et al., 2023)	88.54	55.56	91.32	77.43	60.42	58.68	87.15	83.68	82.64	76.16
CNN-LSTM (Amin et al., 2022)	89.23	72.53	97.23	76.28	82.48	69.15	94.76	86.14	86.1	82.84
EEG-Inception (Zhang et al., 2021)	89.61	80.01	96.17	81.26	83.76	81.2	94.75	98.28	90.5	88.39
CS-CNN (Hu Y. et al., 2023)	91.72	88.48	91.72	88.95	88.31	89.12	89.53	91.78	93.75	90.37
EMPT	93.72	90.03	96.72	93.54	92.61	90.84	95.51	94.11	93.42	93.39

## 4 Discussion

In this study, MI EEG data from ten SCI subjects have been used to constitute a dataset to train a generalized model on the proposed deep learning architecture. To validate the improvement performance, we have analyzed the individual differences of the subjects to enhance the interpretability of the model structure.

### 4.1 Selection of sub-models in the MoE layer

**FIGURE 12:**
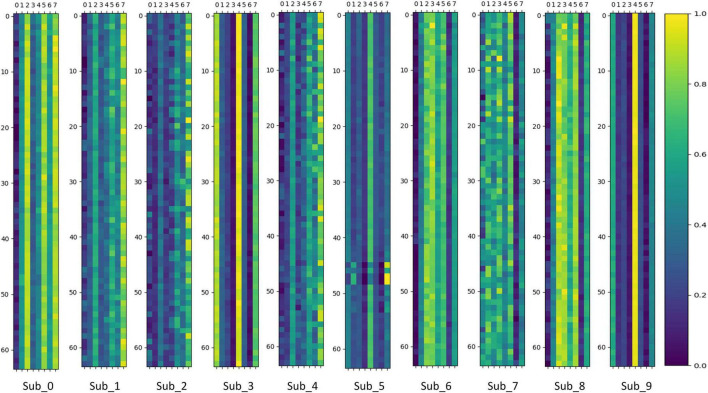
Gating network output values in MoE-TransformerBlock.

**FIGURE 13:**
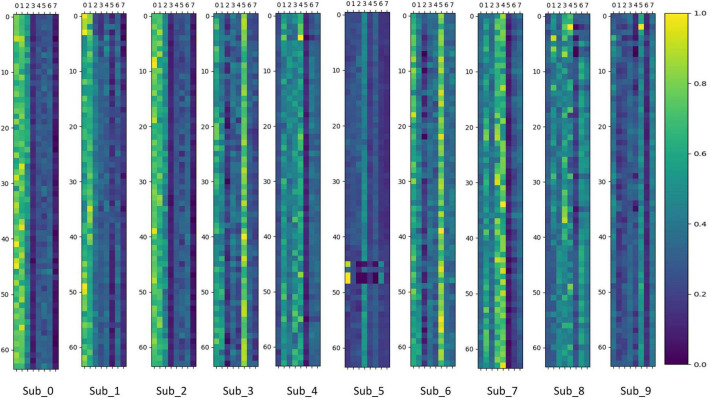
Gating network output values in Prob-MoE-TransformerBlock.

To verify whether the MoE layer in Transformer can effectively perform dynamic sub-model selection for individual subjects and thus achieve model sparsity, this paper visualizes and analyzes the output values of the gating network in the MoE layer. The gating network output values are stacked and averaged according to the number of experiments performed on individual subjects. The results of the visualization of gating network output values in the MoE-TransformerBlock and MoE-Prob-TransformerBlock are shown in Figures 12, 13. The horizontal axis of Figures 12, 13 shows the eight gated values output from the gated network in MoE, and the vertical axis shows the 64 channels of EEG data, with each matrix averaged from the full MI data for a single subject.

We can infer that the gating network responds differently to various subjects, enabling it to assign appropriate gate values to different expert sub-models. Consequently, the MoE layer produces feature vectors that are conducive to the downstream task. For comparison, the MoE layer in MoE-TransformerBlock responds more to the individual differences of subjects while the MoE layer in Prob-MoE-TransformerBlock responds less. This phenomenon shows the fact that the shallow neural network structure is used by the model to extract useful features, which needs to rely on the corresponding linear mapping for different subjects to output feature vectors with low individual differences but with category commonality. In contrast, the features received by the MoE layer in Prob-MoE-TransformerBlock contain fewer individual differences, so the visualization of the gate values turns out to be more similar. In addition, the three channels in Sub_5 behave inconsistently with the performance of other channel gating values, and these three channels are located in similar brain regions. Given that the dataset utilized in this study comprised SCI patients, it is plausible to expect stronger individual variations in brain activity during motor imagery. The phenomenon of gating values behaving differently is due to the fact that MoE provides a different mapping for Sub_5 activity on these three channels than on the other channels, and the differences in activity on the channels may be smaller, but MoE’s mapping decision still made a larger change, which reflects the effectiveness of MoE.

### 4.2 Channel selection situation for ProbSparse self-attention

**FIGURE 14:**
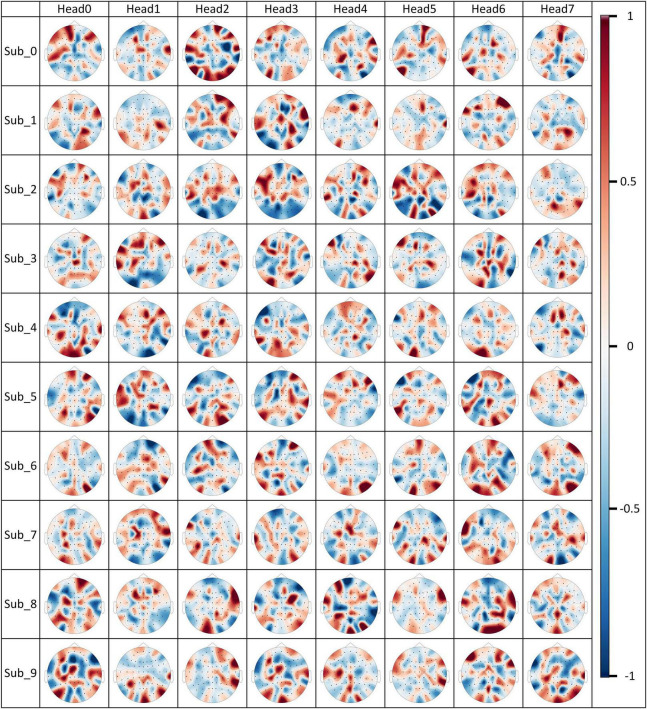
Channel selection for ProbSparse self-attention.

The channel selection situation of ProbSparse self-attention is visualized to observe how the EEG channels have been selected, and the visualization results are shown in Figure 14.

The *M*(*Q*_*i*_,*K*) values computed by individual attention heads on the MI data of a subject have been normalized and are displayed in each subplot of Figure 14. The red color represents a larger *M*(*Q*_*i*_,*K*) value, i.e., it means that the probability that the features of the channel are retained is higher, and vice versa when the location of the channel is in blue color, the probability that the features of the channel are retained is lower. By visualizing the result, we can clearly observe that ProbSparse self-attention is able to select differentiated channel retention schemes in each attention head to generate feature information containing different brain activities. It is important to note that the content of Figure 14 does not fully represent the brain activity situation, although valid brain activity information is retained. Since channel features have already been weighted in the shallow layers of the attentional mechanism, in the deeper layers of the model, the features of a particular channel actually contain a large amount of information about brain activity in other brain regions. The fact that ProbSparse self-attention considers a particular channel in the input features to be worthy of being retained may be an indication that a large number of features of brain activity associated with that channel should be retained and not just that the information about this channel in the raw EEG signal is absolutely important.

## 5 Conclusion

In this study, the EMPT structure is proposed for the classification and identification of EEG signals for MI in SCI patients, and better results have been achieved. This study validates the usability of the MoE module and the ProbSparse self-attention mechanism on EEG signals. The addition of the MoE module and the ProbSparse self-attention mechanism improves the performance of the baseline Transformer model for the EEG classification task and enhances the correctness of the recognition as well as the training stability. The above two improvements are also visualized and analyzed to enhance their interpretability. It is demonstrated that the EMPT structure is very effective in recognizing EEG signals and classifying MI for SCI patients.

## Data availability statement

The datasets presented in this article are not readily available because the article data involves ethical considerations and cannot be disclosed. Requests to access the datasets should be directed to FX, xfz@qlu.edu.cn.

## Ethics statement

The studies involving humans were approved by the Medical Ethics Committee of Qilu Hospital, Cheeloo College of Medicine, Shandong University. The studies were conducted in accordance with the local legislation and institutional requirements. The participants provided their written informed consent to participate in this study.

## Author contributions

ML: Software, Visualization, Writing – original draft. YaL: Data curation, Writing – original draft. WS: Software, Writing – original draft. YiL: Software, Visualization, Writing – original draft. YS: Software, Writing – original draft. QM: Software, Writing – original draft. DW: Data curation, Writing – original draft. FX: Writing – original draft, Writing – review & editing. YZ: Data curation, Writing – review & editing. LZ: Writing – review & editing. JL: Writing – review & editing.
